# Phylogenetic Relationships among the Colobine Monkeys Revisited: New Insights from Analyses of Complete mt Genomes and 44 Nuclear Non-Coding Markers

**DOI:** 10.1371/journal.pone.0036274

**Published:** 2012-04-27

**Authors:** Xiao Ping Wang, Li Yu, Christian Roos, Nelson Ting, Cui Ping Chen, Jing Wang, Ya Ping Zhang

**Affiliations:** 1 Laboratory for Conservation and Utilization of Bio-resource, Yunnan University, Kunming, Yunnan, China; 2 Gene Bank of Primates and Primate Genetics Laboratory, German Primate Center, Göttingen, Germany; 3 Department of Anthropology, University of Oregon, Eugene, Oregon, United States of America; 4 State Key Laboratory of Genetic Resources and Evolution, Kunming Institute of Zoology, Chinese Academy of Sciences, Kunming, Yunnan, China; University of Poitiers, France

## Abstract

**Background:**

Phylogenetic relationships among Asian and African colobine genera have been disputed and are not yet well established. In the present study, we revisit the contentious relationships within the Asian and African Colobinae by analyzing 44 nuclear non-coding genes (>23 kb) and mitochondrial (mt) genome sequences from 14 colobine and 4 non-colobine primates.

**Principal Findings:**

The combined nuclear gene and the mt genome as well as the combined nuclear and mt gene analyses yielded different phylogenetic relationships among colobine genera with the exception of a monophyletic ‘odd-nosed’ group consisting of *Rhinopithecus*, *Pygathrix* and *Nasalis*, and a monophyletic African group consisting of *Colobus* and *Piliocolobus*. The combined nuclear data analyses supported a sister-grouping between *Semnopithecus* and *Trachypithecus*, and between *Presbytis* and the odd-nosed monkey group, as well as a sister-taxon association of *Pygathrix* and *Rhinopithecus* within the odd-nosed monkey group. In contrast, mt genome data analyses revealed that *Semnopithecus* diverged earliest among the Asian colobines and that the odd-nosed monkey group is sister to a *Presbytis* and *Trachypithecus* clade, as well as a close association of *Pygathrix* with *Nasalis*. The relationships among these genera inferred from the analyses of combined nuclear and mt genes, however, varied with the tree-building methods used. Another remarkable finding of the present study is that all of our analyses rejected the recently proposed African colobine paraphyly and hybridization hypothesis and supported reciprocal monophyly of the African and Asian groups.

**Significance:**

The phylogenetic utility of large-scale new non-coding genes was assessed using the Colobinae as a model, We found that these markers were useful for distinguishing nodes resulting from rapid radiation episodes such as the Asian colobine radiation. None of these markers here have previously been used for colobine phylogenetic reconstruction, increasing the spectrum of molecular markers available to mammalian systematics.

## Introduction

The Old World monkeys are comprised of two living subfamilies – the cheek-pouch monkeys (Cercopithecinae) and the leaf-eating monkeys (Colobinae). Although these groups are both the closest living relatives to the apes, research has historically focused on cercopithecine evolution as a model for human evolution. The systematics and evolution of the colobines, on the other hand, has been a relatively neglected topic. The colobinaes consist of 10 genera in two subtribes - the African Colobina (including the genera *Colobus*, *Piliocolobus*, and *Procolobus*) and the Asian Presbytina (including the genera *Pygathrix*, *Rhinopithecus*, *Nasalis*, *Simias*, *Presbytis*, *Trachypithecus*, and *Semnopithecus*) [1]–[4]. Currently, phylogenetic relationships among these genera remain controversial [2], [5]–[11]. The main reason is that colobines represent a typical example of an evolutionary radiation with rapid diversification events that date back to the Middle Miocene about 10–15 million years ago (MYA) [1], [4]. Close to the initial appearance of colobines in the fossil record, nearly all the extant colobine genera diversify from one another within a four million year window [12]–[14]. For this reason, attempts to clarify relationships among these colobine genera have encountered challenges. Given that they share with apes a close relatedness, historically similar distribution in the Old World, and similar timing of diversification events, elucidating colobine evolutionary history can shed light upon the evolution and dispersal of apes (and other mammals) across the Old World, including our own ancestors.

**Figure 1:**
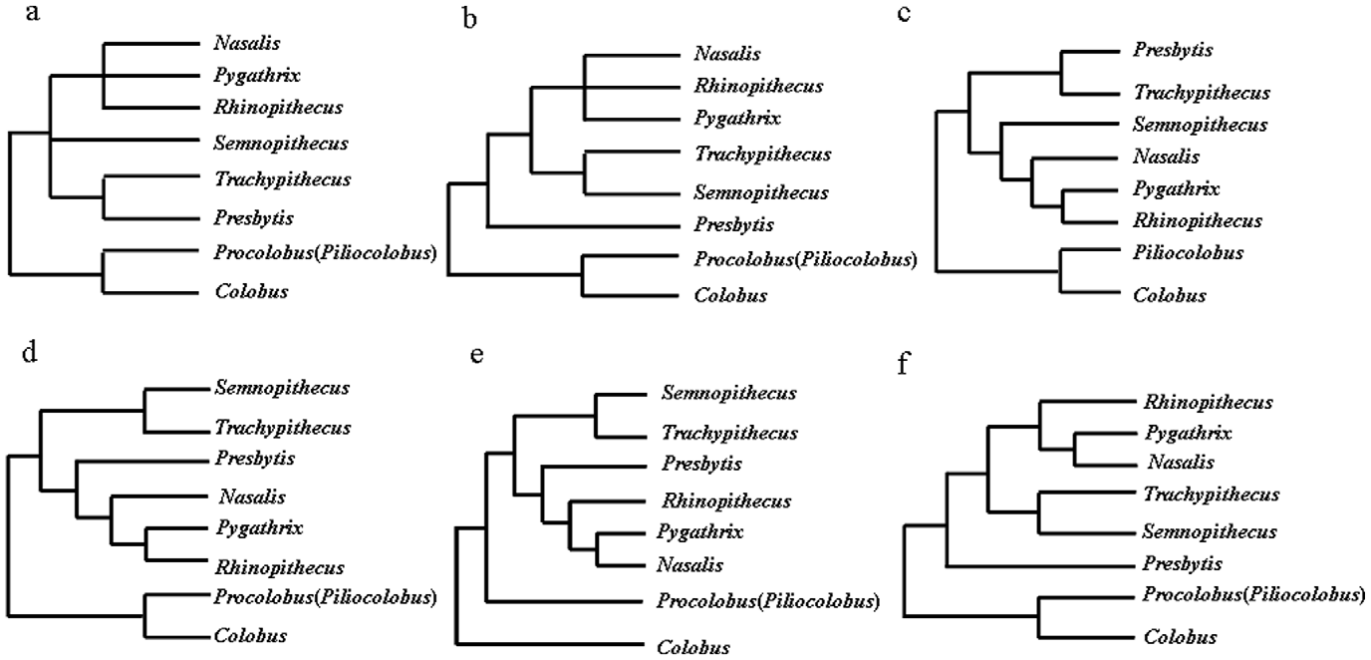
Hypotheses of phylogenetic relationships among Colobine genera. Trees were reconstructed based on (a) 12 protein-coding mt genes [14], (b) fragment of X-chromosome [18] and 54 nuclear genes [12], (c) complete *cytb* gene [16] and 7 mt genes [22], (d) 15 mt genes and 43 nuclear genes [23], (e) 83 mobile elements [13], (f) nuclear genes [13].

Within the Asian colobines, although the monophyly of the odd-nosed monkey group (*Pygathrix*, *Rhinopithecus*, *Nasalis*, and *Simias*) is now widely accepted [1]–[4], [15] and confirmed by genetic data [12]–[14], [16]–[18], monophyly of the langur group (*Trachypithecus*, *Semnopithecus*, and *Presbytis*) is disputed. In fact, recent genetic data provided contradicting relationships among langur genera and the odd-nosed monkey group (see Figure 1) [12]–[14],[16]–[18]. Also, there has been long-standing controversy over the relationships among the genera within the odd-nosed group (see Figure 1) [12]–. For example, compared with earlier investigations that mainly utilized analysis of portions of a single or a small number of mt genes [17], [19]–[21]. Sterner et al. [14] examined 12 mt protein-coding genes of six Asian colobine genera and argued for a sister-group association between *Presbytis* and *Trachypithecus* within the langur group, but failed to resolve the precise relationship among *Presbytis*/*Trachypithecus*, *Semnopithecus*, and the odd-nosed monkey group, as well as the relationships among *Pygathrix*, *Rhinopithecus*, and *Nasalis* within the odd-nosed monkeys (Figure 1a). Ting et al. [18] analyzed a 4,297 bp fragment of the X-chromosome and suggested that within the Asian colobines *Presbytis* diverged earliest, followed by the split between *Trachypithecus*/*Semnopithecus* and the odd-nosed monkey group. However, phylogenetic relationships among *Pygathrix*, *Rhinopithecus*, and *Nasalis* within the odd-nosed monkey group remained unresolved in their analyses (Figure 1b). The same results were also obtained in Perelman et al. [12], which included 54 nuclear genes of 186 primates. In contrast, Chatterjee et al [22] analyzed a 6,138 bp mt fragment and Meyer et al. [16] analyzed a 1.8 kb fragment, and both inferred an earliest divergence of *Presbytis*/*Trachypithecus*, and the close relatedness of *Semnopithecus* and the odd-nosed monkey group (Figure 1c), but they lacked significant support. Through an analysis of 15 mt and 43 nuclear genes, Fabre et al. [23] found support for close relationships between *Trachypithecus* and *Semnopithecus*, and between *Presbytis* and the odd-nosed monkeys (Figure 1d). These same relationships were also recovered Roos et al. [13] from an analysis of 83 mobile elements. However, phylogenetic relationships among *Pygathrix*, *Rhinopithecus*, and *Nasalis* within the odd-nosed monkey group were different between Fabre et al. [23] and Roos et al. [13] (Figure 1e). Intriguingly, in Roos et al.'s [13] nuclear sequence data (∼13 kb) analyses, they also support the former *Semnopithecus-Trachypithecus* clade, but suggest *Presbytis* as sister to the other Asian colobines (Figure 1f). The results from these studies demonstrate that the relationships among Asian colobines remain unresolved, although hybridization has been proposed as a most likely explanation for some of these incongruent relationships [13].

Relationships among the African colobines at the genus-level are not as contentious as there are only 2–3 commonly recognized genera (*Piliocolobus*, *Procolobus* and *Colobus*). Previous studies of this group based on morphology and molecular data suggest that the African colobines represent a monophyletic group [1], [2], [7], [24], [25] that contains a sister-taxon relationship between *Piliocolobus* and *Procolobus* to the exclusion of *Colobus* [26]–[29]. Intriguingly, a recent study by Roos et al. [13] based on mobile elements indicated a closer association of the *Piliocolobus*/*Procolobus* clade to Asian genera than to *Colobus*, a relationship that was not rejected by nuclear sequence data in their study [13]. This finding led them to propose African colobine paraphyly and a hypothesis of ancient hybridization, which challenges the current well-recognized monophyly of the African colobines.

These findings highlight the need to gather and analyze additional sequence data sets in order to unravel the phylogenetic relationships among colobine genera. To this end, we sequenced 44 nuclear non-coding genes comprising a total of >23 kb from 14 colobine and 4 non-colobine primates. These 44 nuclear genes are applied for the first time to study colobine phylogeny. In addition, we also undertook analyses of complete mt genomes from these 18 taxa, including 5 newly determined Asian colobine mt genomes and 13 previously published mt genomes [14]. Our objectives were to: (1) provide further insights into the relationships among the colobine genera, and (2) examine the utility of these genes in the context of colobine phylogeny, with special attention to the previously unexplored 44 nuclear non-coding genes.

## Materials and Methods

### Data Sets

Detailed information on the 44 nuclear non-coding genes (mainly intergenic regions and introns) used for the colobine phylogenetic reconstruction is shown in Table S1. These non-repetitive, non-coding genes were selected from Peng et al. [30], in which 280 genes were screened across primates based on bioinformatic analyses of genome sequences available for human (*Homo sapiens*), common chimpanzee (*Pan troglodytes*) and rhesus macaque (*Macaca mulatta*) (*hg18, panTro2, rheMac2*).

Our sampling includes most of the commonly recognized extant colobine genera except for *Simias* and *Procolobus*. We were unable to obtain biomaterials for these taxa, but previous studies of both morphology and genetics have established that *Simias* is the sister-taxon of *Nasalis* [13], [31], [32] and *Procolobus* is the sister-taxon of (and possibly congeneric to) *Piliocolobus* [13], [18], [25], [27] (Table 1). We also follow the classification of Brandon-Jones et al. [3] in assigning *Trachypithecus johnii* and *Trachypithecus vetulus* to the genus *Semnopithecus*, which morphological and molecular studies have supported [3], [17]. For each of the 14 colobine species sampled, total genomic DNA was isolated from blood or frozen tissues using a standard proteinase K or phenol/chloroform extraction [33]. To amplify these non-coding genes, a “touch-down” PCR amplification was carried out using the following parameters with the primer pairs of Peng et al. [30]: 95°C hot start (3 min), 20 cycles of 94°C denaturation (1 min), 60-40°C annealing (1 min), 72°C extension (1 min), and finally 15 cycles of 94°C denaturation (1 min), 55°C annealing (1 min), 72°C extension (1 min). The amplified DNA fragments were purified and sequenced in both directions with an ABI PRISM™ 3700 DNA or 3130xL sequencer following the manufacturer's protocol.

**Table 1:** Species used in this study.

Genus	Species	Common name	Sample Source	MT genomes	Nuclear genes
*Pygathrix*	*P nemaeus*	Douc langur	Vietnam	NC_008220 [14]	JN103440-JN104028
*Rhinopithecus*	*R roxellana*	Sichuan snub-nosed monkey	Gansu Province, China	NC_008218 [14]	JN103440-JN104028
	*R bieti*	Yunnan snub-nosed monkey	Yunnan Province, China	HM125579 [71]	JN103440-JN104028
	*R avunculus*	Tonkin snub-nosed monkey	Vietnam	HM125578 [71]	JN103440-JN104028
*Trachypithecus*	*T hatinhensis*	Hatinh langur	Vietnam	HQ149046(this study)	JN103440-JN104028
	*T germaini*	Germain's silver langur	Vietnam	HQ149047(this study)	JN103440-JN104028
	*T shortridgei*	Shortridge's langur	Sino-Burmese border area	HQ149048(this study)	JN103440-JN104028
*Nasalis*	*N larvatus*	Proboscis monkey	Borneo	NC_008216 [14]	JN103440-JN104028
*Presbytis*	*P melalophos*	Mitered leaf monkey	Sumatra	NC_008217 [14]	JN103440-JN104028
*Semnopithecus*	*S entellus*	Hanuman langur	India	NC_008215 [14]	JN103440-JN104028
	*S johnii*	Nilgiri Langur	Sri Lanka	HQ149050(this study)	JN103440-JN104028
	*S vetulus*	Purple-faced langur	Sri Lanka	HQ149049(this study)	JN103440-JN104028
*Colobus*	*C guereza*	Eastern black and white colobus	zoo specimen	NC_006901 [14]	
	*C angolensis*	Angolan black-and white colobus	zoo specimen		JN103440-JN104028
*Piliocolobus*	*P badius*	Western red colobus	Sierra Leone (MT)		
			Gambia (nuclear)	NC_008219 [14]	JN103440-JN104028
*Macaca*	*M sylvanus*	Barbary macaque		AJ309865 [30]	*rheMac2*
*Pongo*	*P abelli*	Sumatra orangutan		X97707 [30]	*ponAbe2*
*Pan*	*P troglodytes*	Chimpanzee		D38113 [30]	*panTro2*
*Homo*	*H sapiens*	Human		X93334 [30]	*hg18*

The complete mt genome sequences from five Asian colobine species (*Semnopithecus johnii*, *S. vetulus*, *Trachypithecus hatinhensis*, *T. germaini*, *T. shortridgei*) were newly determined here. The mt genome sequences were amplified using the LA PCR™ Kit from Takara Biotechnology Co., Ltd and 10 universal PCR primers (Table S2). Amplification was performed using 32 cycles of 10 sec at 97°C, 5.5 min at 58°C to 68°C, with an initial step of 1.5 min at 94°C and a final step of 10 min at 72°C. Long-Range PCR products, each with a size of ∼4000 bp, were sequenced in both directions using a primer walking strategy. Sequencing was performed in an ABI PRISM™ 3700 DNA sequencer following the manufacturer's protocols. Primer sequence information is available upon request. Where necessary, PCR products were cloned into the PMD18-T Vector and transformed into ultracompetent *E. coli* cells (TaKaRa Biotechnology Co., Ltd. Dalian, China) in order to resolve the difficulty of direct sequencing of control regions arising from long tandem repeats. Five positive clones per ligation reaction were sequenced. Mt sequences obtained were checked to ensure that they did not include nuclear copies of mtDNA-like pseudogenes (numts), as indicated by the fact that the amino acid sequences of protein-coding genes did not possess premature stop codons or frameshifting insertions/deletions. Also, long-range amplifications are less likely to amplify numts, and we assembled the PCR amplifications to ensure that they formed a circular molecule.

Nuclear and mt genome data from 4 non-colobine primates, i.e., human (*H. sapiens*), common chimpanzee (*P. troglodytes*), rhesus macaque (*M. mulatta*), and orangutan (*Pongo abelii*), were also included in the analyses. Their nuclear and mt genome sequences were downloaded from GenBank (for accession numbers see Table 1).

### Alignments and Sequence Characterizations

Sequences were aligned using Muscle 3.8.31 [34] under the default settings. All 44 genes were analyzed separately and in a combined data set. The mt sequences were divided into five data sets: (1) all 13 protein-coding genes combined, (2) 12S and 16S rRNA genes combined, (3) all 22 tRNA genes combined, (4) control region (CR), (5) tRNAs, rRNAs, CR, and protein-coding genes combined. In the analyses of rRNAs (alignment 2) and tRNAs (alignment 3), the data were also partitioned into single-strand stem and base-paired loops based on the models of Gutell et al. [35] and Springer and Douzery [36]. In addition, the 44 nuclear genes and mt genomes were combined into one alignment. Although arguments can be made against combining genomic regions that possibly have different histories, a combined approach (of all nuclear regions and of nuclear and mitochondrial regions) is thought to detect the phylogenetic signal that is most prevalent across the genome, which is also most likely to represent the species tree [37]. Respective alignments are available upon the authors' request.

Pairwise comparisons and sequence characterizations were estimated using MEGA 4.0 [38].

### Phylogenetic Analyses

Phylogenetic analyses of the individual nuclear non-coding genes and mt alignments 1–4, were performed using PAUP* 4.0b10 [39] for maximum-parsimony [MP] and maximum-likelihood [ML] analyses. MrBayes 3.1.2 [40] was used for the Bayesian inference. We used three hominoid species (*Homo*, *Pan*, *Pongo*) for outgroup rooting in all analyses. In MP analyses, a heuristic search was performed with tree-bisection-reconnection (TBR) branch swapping, random addition of taxa, and 1000 replicates per search. Only one of the best trees found during branch swapping was saved. In ML analyses, the best-fit models of sequence evolution were selected using the Akaike Information Criterion (AIC) [41], [42] with Modeltest 3.7 [43]. The chosen models (see Table 2) and their parameters were used to infer ML trees with the heuristic algorithm, 10 random-addition sequence replicates, and TBR branch swapping. The tree reliability under ML analysis was assessed using a bootstrap analysis of 100 replicates [44]. In Bayesian inference, each Metropolis-coupled Markov chain Monte Carlo (MCMC) run for all individual genes employed the model selected by Modeltest for that gene, or the nearest model to that model that could be implemented in MrBayes. Three heated chains and a single cold chain were used in all MCMC analyses and run for 2 million generations. Three simultaneous independent runs were performed. Trees were sampled every 100 generations. The average standard deviation of split frequencies was close to 0.001 when the runs were finished. The first 25% of the trees were discarded as burn-in. A 50% majority-rule consensus of post burn-in trees was constructed to summarize the posterior probability (PP) for each branch.

**Table 2:** Characterization of nuclear non-coding and mt genes examined in the present study.

Sequence type	Fragment name	Aligned length	Parsimony-informative sites	Best fit model	Among-site Rate Variation		Pairwise Distance(%)
					I	α	
Nuclear genes	*chr1-4*	462	30	K81+G	0	0.6033	3.10
	*chr1-6*	567	36	GTR	0	0	3.40
	*chr2-1*	504	32	K80	0	0	3.10
	*chr2-8*	413	28	HKY+G	0	0.6264	3.10
	*chr3-2*	533	28	HKY+G	0	0.4657	2.50
	*chr3-5*	337	33	TVM+I	0.4589	0	5.10
	*chr4-2*	486	26	HKY+G	0	1.3216	5.40
	*chr4-7*	492	34	HKY	0	0	2.90
	*chr5-6*	534	41	HKY	0	0.9808	3.60
	*chr5-8*	480	39	TIM	0	0	3.80
	*chr6-5*	456	43	TIM+G	0	0.7324	4.10
	*chr6-6*	367	23	HKY+I	0.5102	0	3.30
	*chr7-6*	514	49	K81uf	0	0	5.20
	*chr8-1*	577	51	HKY+G	0	0.7446	4.50
	*chr8-2*	526	29	HKY	0	0	2.80
	*chr9-5*	522	26	GTR+G	0	1.1437	3.20
	*chr10-1*	503	46	TrN+I	0.4591	0	4.20
	*chr10-5*	498	9	TVMef+I	0.6203	0	1.40
	*chr11-2*	522	30	TIM+G	0	0.5405	4.00
	*chr12-1*	586	44	TVMef+I	0.4245	0	3.50
	*chr12-2*	439	30	HKY	0	0	3.20
	*chr13-3*	401	23	HKY	0	0	3.00
	*chr13-6*	472	31	K81uf	0	0	3.30
	*chr15-1*	862	302	HKY	0	0	22.60
	*chr15-3*	398	21	TrN	0	0	2.50
	*chr17-4*	788	141	TVMef+G	0	1.1752	8.60
	*chr17-8*	497	44	TrN+G	0	0.9342	4.70
	*chr18-4*	504	30	HKY+I	0.6339	0	2.60
	*chr19-1*	550	55	HKY+I	0.5819	0	4.50
	*chr19-5*	458	45	HKY+I	0.5243	0	3.70
	*chr20-4*	588	58	K81uf+I	0.5311	0	4.00
	*chr20-5*	457	32	HKY+G	0	0.8146	3.70
	*ENC5*	641	39	HKY+I	0.4338	0	3.00
	*ENC14*	539	30	GTR	0	0	2.50
	*ENC15*	868	49	HKY	0	0	3.00
	*ENC19*	530	29	TVM	0	0	2.90
	*ENC25*	401	32	HKY+G	0	0.7782	3.90
	*ENC35*	548	34	TVM+I	0.4981	0	2.60
	*X2*	565	37	HKY+G	0	0.457	3.60
	*X5*	510	38	HKY+G	0	1.04	3.50
	*X37*	598	56	TVM+G	0	0.4556	4.20
	*X45*	490	24	GTR+G	0	1.2507	3.00
	*X61*	602	39	TVM+G	0	0.8788	2.90
	*X65*	549	32	K81uf+G	0	0.603	2.70
	Combined	23134	1951	TVM+G	0	0.7034	3.90
Mt genes	*ND1*	957	337	GTR+I+G	0.4389	1.2434	19.00
	*ND2*	1044	426	TrN+I+G	0.3765	1.3323	22.80
	*COX1*	1545	501	TVM+I+G	0.5698	1.9262	16.90
	*COX2*	684	227	HKY+I+G	0.5132	1.2176	17.10
	*ATP8*	211	104	TrN+I+G	0.2533	1.1544	28.80
	*ATP6*	681	281	TIM+I+G	0.3344	0.964	23.60
	*COX3*	784	274	HKY+I+G	0.5063	1.237	18.60
	*ND3*	346	144	K81uf+I+G	0.4127	2.2742	23.40
	*ND4L*	297	108	K81uf+I+G	0.4101	1.204	19.80
	*ND4*	1378	532	TrN+I+G	0.4155	1.2687	21.00
	*ND5*	1806	709	TIM+I+G	0.3871	1.2705	22.40
	*ND6*	528	181	TrN+I+G	0.3847	0.7419	18.90
	*CYTB*	1135	430	HKY+I+G	0.4267	1.1196	20.70
	*12SrRNA*	961	223	GTR+I+G	0.4386	0.6382	10.90
	*16SrRNA*	1582	375	GTR+I+G	0.4575	0.7409	12.70
	*tRNA*	1573	377	GTR+I+G	0.3032	0.4305	12.10
	*D-loop*	1015	415	TVM+I+G	0.2362	0.81	24.80
	Combined	16527	5644	GTR+I+G	0.4328	1.0995	18.50

Note: Ti = Transition; Tv = Transversion; I = Proportion of invariable sites; α = Gamma distribution shape parameter.

In addition to individual analyses, phylogenetic reconstructions were performed on the combined nuclear dataset and the combined mt genome dataset (mt alignment 5) as well as the combined nuclear and mt genome dataset. We used PAUP* for the MP analysis, the RAxML online web server [45] for a partitioned ML analysis with a GTR model, and MrBayes and PhyloBayes for Bayesian analyses [46]. For each combined dataset, we identified model partitions based on partitioning matrices by gene. That is, in the analysis of the combined nuclear data set, each nuclear non-coding gene was considered a different partition, and in the combined mt data set, each of the 13 individual protein-coding genes, all tRNAs, and each of the two rRNA genes were considered to be different partitions. Based on the selected models using the AIC [41], [42] as mentioned above for individual analyses (see Table 2), we assigned a separate substitution model for each of the data partitions in the MrBayes analysis. Three heated chains and a single cold chain were used in all MCMC analyses and run for 5 million generations, sampling trees every 100 generations. The average standard deviation of split frequencies was close to 0.001 when the run ended. The first 25% of trees were discarded as burn-in. A 50% majority-rule consensus of post burn-in trees was constructed to summarize PPs for each branch. In addition, the site-heterogeneous mixture model CAT-GTR was used for the above three combined datasets in PhyloBayes analysis [47] with two independent (MCMC) chains. Compared to other phylogenetic MCMC samplers, the main distinguishing feature of PhyloBayes is the underlying probabilistic model, CAT [48]. CAT is a mixture model especially devised to account for site-specific features of sequence evolution. It is particularly well suited for large multigene alignments. To check for convergence, the program bpcomp [49] was used to compare the bipartitions between the two runs. With a burn-in of 1000 and taking every two trees, the largest discrepancy (maxdiff) between the bipartitions was less than 0.1.

### Testing Potential Tree Incongruence

The incongruence among different tree topologies was evaluated using the Shimodaira-Hasegawa (SH) test [50] and the approximately unbiased (AU) test [51], as implemented in the CONSELV0.1i program [52] with default scaling and replicate values. The site-wise log-likelihood values were estimated by PAUP*.

## Results

### Characteristics of the Nuclear Non-Coding Data and Mt Genomes

The general characteristics of the nuclear non-coding data and mt genomes are summarized in Table 2. The 44 nuclear non-coding genes of 18 species varied in length from 337 bp (*chr3-5*) to 862 bp (*ENC15*) aligned positions. The numbers of parsimony-informative sites range from 9 (1.81%) (*chr10-5*) to 302 (35.03%) (*chr15-1*). The combined alignment of the 44 non-coding genes was comprised of 23,134 bp, 1,951 bp (8.43%) of which are parsimony-informative sites. The nuclear sequence divergence ranged from 1.40% (*chr10-5*) to 22.60% (*chr15-1*), and averaged 4.01%.

The complete mt genomes range from 16,499–16,648 bp in size. Length differences were largely due to the variation in copy number of tandem repeat sequences in the conserved sequence block (CSB) domains of the mt control region. All genomes shared not only 13 protein-coding genes, 22 tRNAs, 2 rRNAs, and a control region, but also the same gene order. The mt genome sequence divergence ranged from 16.90% (*COX1*) to 28.80% (*ATP8*) for the protein-coding dataset (average 21.00%), from 10.90% (12S rRNA) to 12.70% (16S rRNA) for the rRNA dataset (average 11.80%), 12.10% for the 22 tRNA dataset, 24.80% for the control region, and 18.50% for the complete dataset.

### Phylogenetic Inference

**Figure 2:**
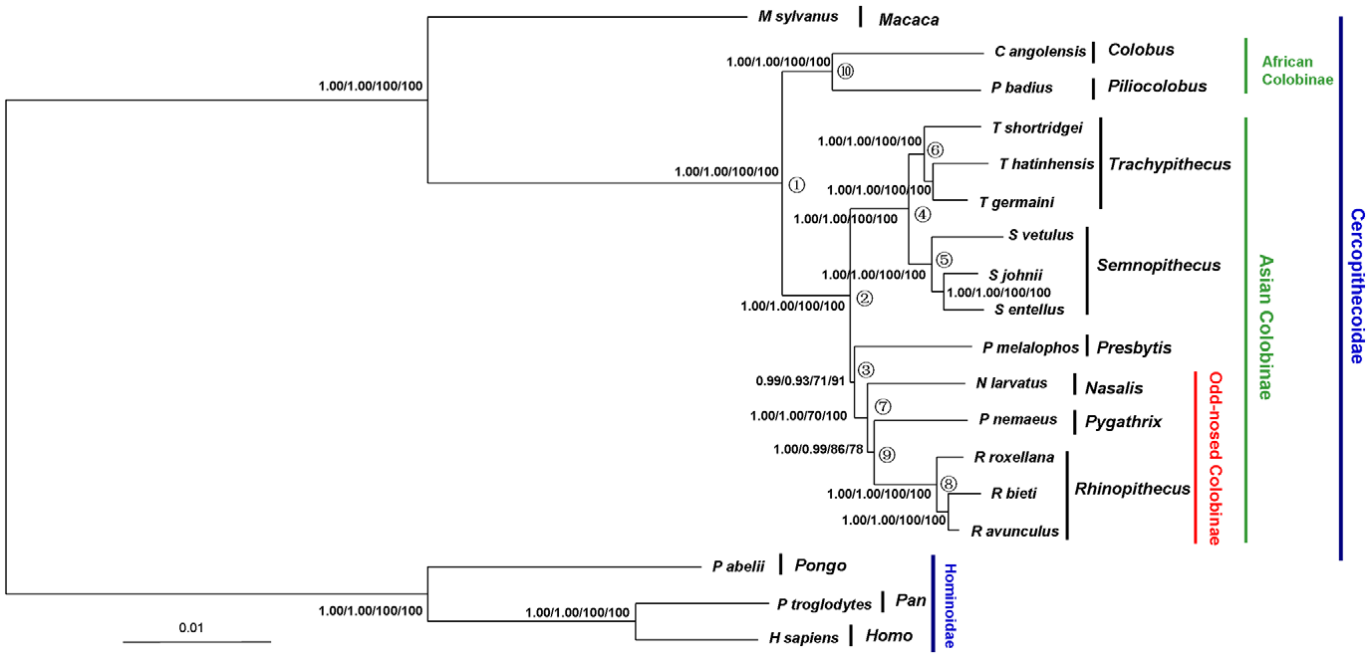
Phylogenetic tree inferred from the combined 44 nuclear non-coding genes. The nodal supports (Bayesian PP/PhyloBayes PP/ML BS/MP BS) are shown above the nodes. Node numbers in the tree indicate the nodes that were used in divergence time estimations and phylogenetic performance evaluation.

Although individual nuclear gene analyses produced incongruent topologies with low levels of nodal support (Figure S1), possibly due to limited phylogenetic information harbored in a single marker, the analyses of the combined nuclear data set using four different tree-building methods (MP, ML, Bayesian and PhyloBayes) yielded an identical, well-resolved tree topology with strong support for most nodes (Figure 2). All analyses divided colobines into reciprocally monophyletic Asian and African clades (MP BS = 100%; ML BS = 100%; Bayesian PP = 1.00; PhyloBayes PP = 1.00). African paraphyly as suggested by Roos et al. [13] was rejected by our nuclear data (P<0.05). The Asian colobines were grouped into two clades. Clade 1 included *Presbytis* and the odd-nosed monkey group (MP BS = 91%; ML BS = 71%; Bayesian PP = 0.99; PhyloBayes PP = 0.93), and Clade 2 included *Semnopithecus* and *Trachypithecus* (MP BS = 100%; ML BS = 100%; Bayesian PP = 1.00; PhyloBayes PP = 1.00). Within the monophyletic odd-nosed monkey group (MP BS = 100%; ML BS = 70%; Bayesian PP = 1.00; PhyloBayes PP = 1.00), *Rhinopithecus* and *Pygathrix* clustered together (MP BS = 78%; ML BS = 86%; Bayesian PP = 1.00; PhyloBayes PP = 0.99) to the exclusion of *Nasalis*. The other two alternative phylogenetic relationships among *Rhinopithecus*, *Nasalis* and *Pygathrix*, however, were not rejected by our nuclear data (P>0.05). In addition, the alternative placement of *Presbytis* as either the sister taxon to all other Asian colobines or to the *Semnopithecus*/*Trachypithecus* clade was not rejected (P>0.05).

**Figure 3:**
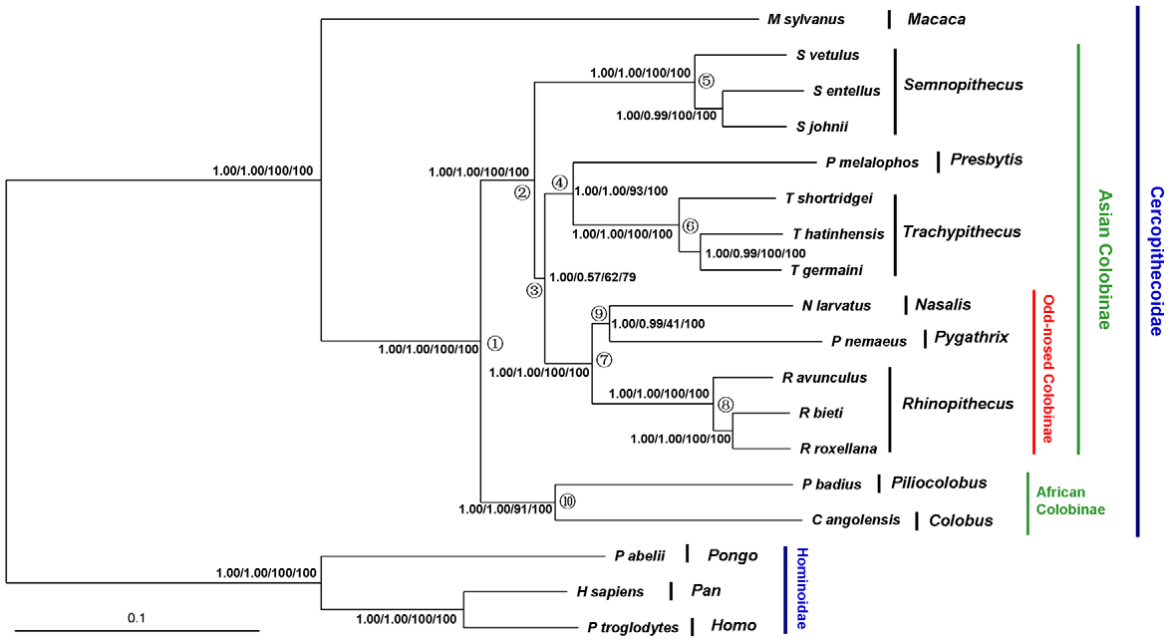
Phylogenetic tree inferred from the mt genome sequences. The nodal supports (Bayesian PP/PhyloBayes PP/ML BS/MP BS) are shown above the nodes. Node numbers in the tree indicate the nodes that were used in divergence time estimations and phylogenetic performance evaluation.

For mt gene analyses, the combined rRNA and combined tRNA data demonstrated low resolving power for phylogenetic inference compared to the combined protein-coding gene analyses and the control region analysis (see Figure S2). In comparison, the complete mt genome-based analyses, irrespective of the tree-building method that was used, produced a well-resolved and well-supported tree (Figure 3), with the tree topology being identical to those of Bayesian, PhyloBayes and ML analyses of the combined protein-coding gene analysis (Figure S2). The analyses divided colobines into reciprocally monophyletic Asian and African clades (MP BS = 100%; ML BS = 100%; Bayesian PP = 1.00; PhyloBayes PP = 1.00). African paraphyly was rejected by our mt data (P<0.05). Within the Asian Colobinae, *Semnopithecus* diverged first (MP BS = 79%; ML BS = 62%; Bayesian PP = 1.00; PhyloBayes PP = 0.57), and the odd-nosed monkey group was sister to *Presbytis* and *Trachypithecus*. Monophyly of these respective clades was strongly supported (MP BS = 100%; ML BS = 93%; Bayesian PP = 1.00; PhyloBayes PP = 1.00). Within the odd-nosed monkey group, *Pygathrix* and *Nasalis* clustered together to the exclusion of *Rhinopithecus* (MP BS = 100%; ML BS = 41%; Bayesian PP = 1.00; PhyloBayes PP = 0.99), but alternative relationships were not rejected (P>0.05). Although *Semnopithecus* is suggested as the first lineage to diverge, a clade together with the odd-nosed monkey group, which was indicated in previous mt studies, is not rejected (P>0.05).

**Figure 4:**
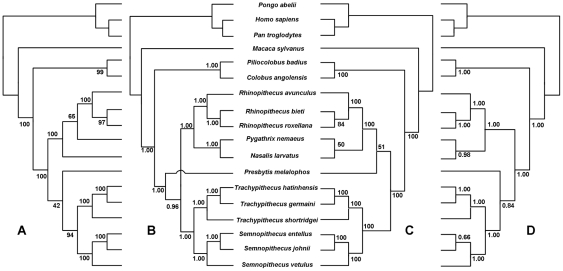
Phylogenetic trees inferred from the combined nuclear and mt dataset. Trees are reconstructed using MP (A), Bayesian Inference (B), ML (C) and PhyloBayes (D). The nodal support values are shown above the nodes, respectively.

For the combined nuclear genes and mt genome dataset analyses, four different tree topologies were produced using four tree-building methods (MP, ML, Bayesian, PhyloBayes) (Figure 4). These tree topologies all support the monophyly of the Asian and African clades (MP BS = 100%; ML BS = 100%; Bayesian PP = 1.00; PhyloBayes PP = 1.00) and the monophyly of the odd-nosed monkey group (MP BS = 100%; ML BS = 100%; Bayesian PP = 1.00; PhyloBayes PP = 1.00), as well as the sister-group relationship between *Semnopithecus* and *Trachypithecus* (MP BS = 94%; ML BS = 100%; Bayesian PP = 1.00; PhyloBayes PP = 1.00). In comparison, the relationships among *Pygathrix*, *Nasalis* and *Rhinopithecus* within the odd-nosed monkey group and the placement of *Presbytis* within the Asian clade varied with the analytic methods used.

### Assessing the Performance of Individual Nuclear and Mt Genes

The use of such a large nuclear DNA dataset and mt genome sequences from Asian colobines provides the opportunity to not only infer a colobine phylogeny but to evaluate the phylogenetic performance of the individual nuclear and mt genes as well.

The same four tree-building methods as described above were also performed on the 44 nuclear non-coding genes, 13 mt protein-coding and 2 rRNA genes individually (see Figure S1 and Figure S3; only Bayesian analyses are shown). As can be seen from the resulting phylogeny of these single-gene analyses, including those from tRNA genes and the control region (Figure S2), the relationships among Asian colobine genera were either not recovered at all or varied considerably with little or no nodal support.

We also assessed the phylogenetic utility of individual non-coding genes and mt genes in their ability to resolve the inter-generic relationships of the Colobinae by counting the number of congruent nodes between the individual phylogenies and the combined gene trees (Table 3). In the individual nuclear gene analyses, the *chr12-1* gene recovered the highest number of nodes (8) congruent with the combined nuclear gene tree. The *chr1-4*, *chr6-6*, *chr12-2*, *chr15-1 chr19-1*, *ENC25*, *chr15-3*, *ENC14*, and *X37* genes showed the fewest congruent nodes with the combined nuclear gene tree and thus had the lowest phylogenetic performance. In regard to the mt gene analyses, we observed that the *COX1* gene recovered all 10 nodes of the mt genome tree. Ranking the single mt gene shows that the *COX1*, *ND2* and *ND4* genes are better indicators of colobine phylogeny at the genus level than are other genes, such as the *ATP8*, *ND3* and *ND4L* genes. This result agrees broadly with previous conclusions about the rough classification of mt genes into good, medium, and poor performance categories [53]–[59] (Table S3). In summary, the assessment of the phylogenetic utility and limits of these individual nuclear and mt genes make it possible to preselect subsets of genes for future molecular studies of vertebrate phylogeny.

**Table 3:** Phylogenetic performance of nuclear and mt genes. Node numbers correspond to those indicated in Figure 2 and 3.

Gene^a^		no. congruent branches (BP>0.95)	no. congruent branches (BP<0.95)	total no. congruent branches	Node^b^									
					1	2	3	4	5	6	7	8	9	10
Nuclear genes	*chr12-1*	7	1	8	*	*		*	*	#	*	*		*
	*X61*	6	2	8	#	*		*	*	#	*	*		*
	*chr17-4*	6	1	7	*	*	*	*				*	#	*
	*chr20-4*	4	3	7	*	*		#			#	#	*	*
	*chr5-6*	5	1	6	*	*		#			*	*		*
	*chr20-5*	4	2	6	#	*		*			*	#		*
	*ENC35*	5	1	6	#	*		*	*			*		*
	*ENC15*	0	5	5	#		#			#	#			#
	*chr1-6*	1	3	4				#	#			*	#	
	*chr2-8*	1	3	4	#							*	#	#
	*chr3-5*	2	2	4	#			#				*		*
	*chr6-5*	2	2	4				*	*	#		#		
	*chr17-8*	2	2	4		*		*	#			#		
	*X45*	2	2	4				*				*	#	#
	*chr4-2*	1	2	3				#				*		#
	*chr7-6*	1	2	3				#				*		#
	*chr8-1*	2	1	3						*		*		#
	*chr9-5*	0	3	3							#	#		#
	*chr13-3*	2	1	3		*				*		#		
	*chr18-4*	1	2	3		*	#	#						
	*ENC5*	2	1	3		#		*				*		
	*ENC19*	0	3	3							#	#		#
	*X5*	2	1	3				#				*		*
	*X65*	1	2	3				#			*			#
	*chr2-1*	0	2	2					#			#		
	*chr3-2*	1	1	2								#		*
	*chr4-7*	2	0	2				*						*
	*chr5-8*	0	2	2							#	#		
	*chr8-2*	1	1	2						#		*		
	*chr10-1*	2	0	2						*		*		
	*chr10-5*	0	2	2						#		#		
	*chr11-2*	1	1	2							#	*		
	*chr13-6*	0	2	2				#		#				
	*chr19-5*	2	0	2				*				*		
	*X2*	0	2	2						#				#
	*chr1-4*	1	0	1				*						
	*chr6-6*	0	1	1						#				
	*chr12-2*	0	1	1					#					
	*chr15-1*	1	0	1								*		
	*chr19-1*	1	0	1								*		
	*ENC25*	0	1	1								#		
	*chr15-3*	0	0	0										
	*ENC14*	0	0	0										
	*X37*	0	0	0										
Mt genes	*COX1*	9	1	10	*	*	*	*	*	*	*	*	#	*
	*ND2*	8	1	9	*	*		*	*	*	*	*	#	*
	*ND4*	7	2	9	*	*	#	#	*	*	*	*		*
	*ATP6*	6	2	8	*	*			*	*	#	*	#	*
	*ND5*	8	0	8	*	*			*	*	*	*	*	*
	*16SrRNA*	7	1	8	*	*			*	*	*	*	#	*
	*tRNA*	7	1	8	*	*			*	*	*	*	*	#
	*D-loop*	8	0	8	*			*	*	*	*	*	*	*
	*ND1*	6	1	7	*			*	*	*	#	*		*
	*COX2*	3	4	7	*	#	#		*	*			#	#
	*COX3*	6	1	7	*				*	*	*	*	#	*
	*ND6*	6	1	7	#	*			*	*	*	*		*
	*CYTB*	6	0	6	*				*	*	*	*		*
	*12SrRNA*	6	0	6	*	*			*	*		*		*
	*ND4L*	3	2	5	*				*	#	#	*		
	*ND3*	2	2	4	#				*			*		#
	*ATP8*	1	1	2					*	#				

a genes are ranked by the total number of congruent branches in the combined topologies.

b there are 10 nodes in total indicated in the combined nuclear gene tree (Figure 2) and the mt genome tree (Figure 3).

* branches with PP>0.95 congruent in the combined topology.

# branches with PP<0.95 congruent in the combined topology.

## Discussion

Among mammalian phylogenies, those characterized by rapid species radiations have long been a challenging problem in species tree reconstruction [60]. This is among the first studies to utilize data from such large-scale nuclear non-coding genes in the Colobinae, which provides new insights into the phylogenetic resolution of the colobines.

### Phylogeny of the Asian Colobinae

In our study, different phylogenetic relationships among Asian colobine genera were recovered by analyzing combined nuclear data, combined mt genome data, as well as combined nuclear and mt data, except for the consensus of clustering *Rhinopithecus*, *Pygathrix* and *Nasalis* together. This corroborates the prevailing definition of a monophyletic ‘odd-nosed’ group within the Asian Colobinae composed of *Rhinopithecus*, *Pygathrix*, *Nasalis* and *Simias* [61]–[64] and supports previous findings of mitochondrial and nuclear gene tree discordance due to ancient hybridization among the langurs [13], [18].

All analyses of combined nuclear non-coding data (Figure 2), and the ML analyses of combined nuclear and mt data (Figure 4C) supported the sister-grouping between *Semnopithecus* and *Trachypithecus* (BS = 100%; PP = 1.00), and that between *Presbytis* and the odd-nosed monkey group (BS = 51–91%; PP = 0.93–0.99). The recovery of a close affinity between *Semnopithecus* and *Trachypithecus* here is in accordance with previous nuclear analyses [12], [13], [18] and retroposon integration analyses [13], [17] as well as previous combined mt and nuclear dataset analyses [19]. The close relatedness of *Presbytis* and the odd-nosed monkey group, however, is interesting, because this finding disagrees with the Bayesian analyses of combined nuclear and mt data (Figure 4B) (PP = 0.96), as well as those based on previous and recent nuclear sequence analyses where *Presbytis* was placed as the earliest diverging genus among the Asian Colobinae [12], [13], [18], but it is consistent with that from mobile elements analysis [13] and previous combined mt and nuclear dataset analyses [23]. Interestingly, the MP and PhyloBayes analysis of the combined nuclear and mt dataset clusters *Presbytis* with the *Semnopithecus*/*Trachypithecus* clade, supporting the monophyly of the langur group (Figure 4A and D). But this relationship receives low support values. Despite our findings on the placement of *Presbytis*, tree topology tests do not reject alternative placements of this taxon within the Asian colobine tree. More nuclear data need to be collected to elucidate this issue.

The mt genome data analysis yielded a different tree topology (Figure 3). *Semnopithecus* diverged earliest within the Asian colobines and the odd-nosed monkey group was the sister-taxon to a clade uniting *Presbytis* and *Trachypithecus* (BS = 62–79%; PP = 1.00). In previous mt analyses that were based on partial genes and/or less taxonomic sampling, *Semnopithecus* either formed an unresolved polytomy with the other Asian colobine genera [13], [14], [17], [18] or was more closely related to the odd-nosed monkey group [16], [21], [22]. Thus, our mt genome data analysis provides support for a new phylogenetic hypothesis. However, alternative positions of *Semnopithecus* among Asian colobines are not rejected by our mt dataset (P>0.05). Therefore, evidence from additional data is necessary to further elucidate the placement of *Semnopithecus* within the Asian colobine mt gene tree. The association of *Presbytis* with *Trachypithecus* revealed here is consistent with most previous mt studies [14], [16], [18], [22].

In addition, an overview of our phylogenetic results revealed a topological discrepancy for the relationships among *Rhinopithecus*, *Pygathrix* and *Nasalis* within the odd-nosed monkey group. All analyses of combined nuclear data (Figure 2) and the MP analysis of the combined nuclear and mt dataset (Figure 4A) indicated a sister-taxon association between *Pygathrix* and *Rhinopithecus* to the exclusion of *Nasalis* (BS = 65–86%; PP = 0.99–1.00), whereas all analyses of mt genome data and the ML, Bayesian and PhyloBayes analyses of combined nuclear and mt dataset datasets (Figure 3, Figure 4B, C, D) supported a close association of *Pygathrix* with *Nasalis* (BS = 41–100%; PP = 0.98–1.00). The former result is consistent with those inferred from morphological and previous mt gene fragment analyses [5], [16],[19],[20],[31], and the latter result is in agreement with Roos et al. [13], who inferred relationships from analyses of mobile elements, nuclear sequence data, and mt genome sequences. Tree topology tests indicated that the sister-taxon association of *Pygathrix* and *Rhinopithecus* revealed by the nuclear data analyses was not rejected by both our mt dataset and the combined nuclear and mt dataset (P>0.05), and the association of *Pygathrix* with *Nasalis* revealed by the mt dataset was not rejected by both our nuclear data and the combined nuclear and mt dataset (P>0.05). The alternative hypothesis grouping *Nasalis* and *Rhinopithecus* inferred from the mt *cytb* analysis [21] was not recovered here in any of the analyses

### Monophyly of the African Colobinae

Our combined nuclear data and mt genome data as well as the combined nuclear and mt datasets all clustered the two African colobine genera, i.e., *Colobus* and *Piliocolobus*, together with robust support (BS = 91–100%; PP = 1.00) (Figure 2, 3, 4). In the individual analyses of nuclear genes, six genes favored the paraphyly of the African colobines, but only one of them grouped *Piliocolobus* with the Asian colobines with high support (sensu Roos et al. [13]; Figure S1). In addition, the three combined datasets all significantly rejected the grouping of *Piliocolobus* with the Asian colobines (P<0.05), as well as the six individual alternative gene tree topologies (P<0.05). Our study thus supports the traditional view of African colobine monophyly and disagrees with the African colobinae paraphyly hypothesis proposed by Roos et al. [13], in which mitochondrial and nuclear gene tree discordance was explained by female introgression from *Procolobus*/*Piliocolobus* into *Colobus*. It is thus possible that the nuclear genes (three transposable element insertion events) that supported African colobine paraphyly in Roos et al. failed to sort into lineages that represent the species phylogeny. An alternative explanation is that female introgression from *Procolobus*/*Piliocolobus* into *Colobus* did indeed occur, but it was so extensive that very little evidence remains in the nuclear genome. Such minor signal would not be detected in a combined gene analysis.

### Utility of the nuclear non-coding genes and mt genes in phylogenetic analysis of the Colobinae

Several recent studies have indicated that nuclear non-coding genes hold considerable signals for resolution of difficult phylogenies at both shallow and deeper species level hierarchies [59], [65]–[70]. We are among the first to use large-scale nuclear non-coding genes in inferring colobine phylogeny. Our analysis not only brings new perspectives on the phylogenetic relationships among colobine genera, but provides another example demonstrating that nuclear non-coding genes can be an effective data source for reconstructing evolutionary histories in a group that has undergone rapid bursts of speciation.

As can be seen from the results of individual gene analyses, we found that among the 44 non-coding nuclear genes, *chr17-4*, *chr12-1*, *X61* and *chr20-4* provide a higher level of phylogenetic resolution, while *chr1-4*, *chr6-6*, *chr12-2*, *chr15-1 chr19-1*, *ENC25*, *chr15-3*, *ENC14*, and *X37* genes contribute the lowest levels of phylogenetic signal. When ranking single mt genes by their respective contribution to the mt genome tree, we found that some genes, such as *COX1*, *ND2* and *ND4* genes are better indicators of colobine evolutionary relationships than are other genes, such as *ATP8*, *ND3* and *ND4L* genes (Table 3). Our results agree globally with those from previous mt studies of other mammalian groups regarding the rough classification of mt individual genes into good, medium, and poor categories [53]–[59] (Table S3). In all of these studies, *ND2* and *COX1* genes were always included in the good category, whereas *ND4L* and *ATP8* were included in the poor category. In contrast to previous conclusions, the present work indicates that the *CYTB* and *12SrRNA* genes are poor genetic markers for reconstructing the genus-level relationships within the Colobinae. The assessment of phylogenetic values of these nuclear and mt genes makes it possible to preselect subsets of nuclear and mt genes for phylogenetic questions at different taxonomic levels in the case of unavailable genome sequences.

### Reasons for gene tree incongruence

This study raises questions regarding why the gene trees inferred here from different markers sometimes differ from one another and also from those inferred in other studies. The main areas of gene tree incongruence seem to be 1) the interrelations of the langurs between mt and nuclear DNA data (*Presbytis, Semnopithecus, Trachypithecus*), 2) the relationships among the African colobines in the transposable element tree versus trees inferred from mitochondrial and nuclear sequence data, 3) the placement of *Presbytis* in the nuclear DNA tree, 4) the interrelations of the odd-nosed monkeys. This research agrees with previous work [13], [18] in showing that the mitochondrial and nuclear gene tree discordance in the langurs is likely the result of ancient hybridization. Multiple independent nuclear markers now show that *Trachypithecus* and *Semnopithecus* are sister taxa. It is unlikely that these are all the result of incomplete lineage sorting, and it is likely that the mitochondrial lineages sorted prior to the nuclear lineages to represent the original tree (due to the smaller effective population size of the mitochondrial genome). We also believe that the African colobine paraphyly is likely due to incomplete lineage sorting of the genomic regions from where the transposable elements were sampled in Roos et al. [13]. However, it is also possible that these areas of the genome are actually representative of an ancient hybridization event, but the vast majority of the genome no longer carries that signal because hybridization was extensive. These two scenarios of incomplete lineage sorting and extensive hybridization are very difficult to disentangle. The remaining issues of gene tree discord among the colobines found here are likely due to the presence of very short internodes that preclude the capture of sufficient variation that is required to produce well-supported and well-resolved phylogenetic relationships. This is the reason why different methods generated different arrangements and why alternative arrangements are not rejected despite apparent high support. The combination of nuclear and mitochondrial datasets did not overcome this issue, possibly because of drastically different rates of evolution and different population histories. However, it is important to point out that the use of 44 non-coding regions (>23 kb) provided as much resolution, if not more, than the much faster evolving mitochondrial genomes. Thus, we believe that the collection of even more nuclear sequence data, particularly from non-coding regions that are not under purifying selection, will provide even greater resolution to the phylogenetic relationships among the colobines. Regardless, it is now even more apparent that the colobines underwent a very rapid radiation, especially among the Asian taxa, which might have required rapid and successive biogeographic vicariance events that would have affected other taxa in the Late Miocene as well.

## Supporting Information

Figure S1 **Bayesian trees of the individual nuclear non-coding genes.** PPs are presented above nodes.
(TIF)

Figure S2 **Bayesian trees of the mt datasets (1)–(4).** PPs are shown above nodes. Since the resulting tree topologies for the 13 combined protein-coding gene data set differ among the MP/Bayesian/PhyloBayes/ML analyses, trees from all four reconstructions are shown. BS values are shown above nodes.
(TIF)

Figure S3 **Bayesian trees of the individual mt genes.** PPs are shown above nodes.
(TIF)

Table S1 Detailed information of the 44 non-coding genes used in the present study.
(DOC)

Table S2 The universal long-range PCR primer information used for mt genome amplification.
(DOC)

Table S3 Comparison of phylogenetic performance of mt genes between our study and previous studies.
(DOC)
