# Supplementary figures and images for: Phylogenetic Relationships among the Colobine Monkeys Revisited: New Insights from Analyses of Complete mt Genomes and 44 Nuclear Non-Coding Markers
Source: PLoS One. 2012 Apr 27;7(4):e36274. doi: 10.1371/journal.pone.0036274 (PMC3338693; doi:10.1371/journal.pone.0036274)

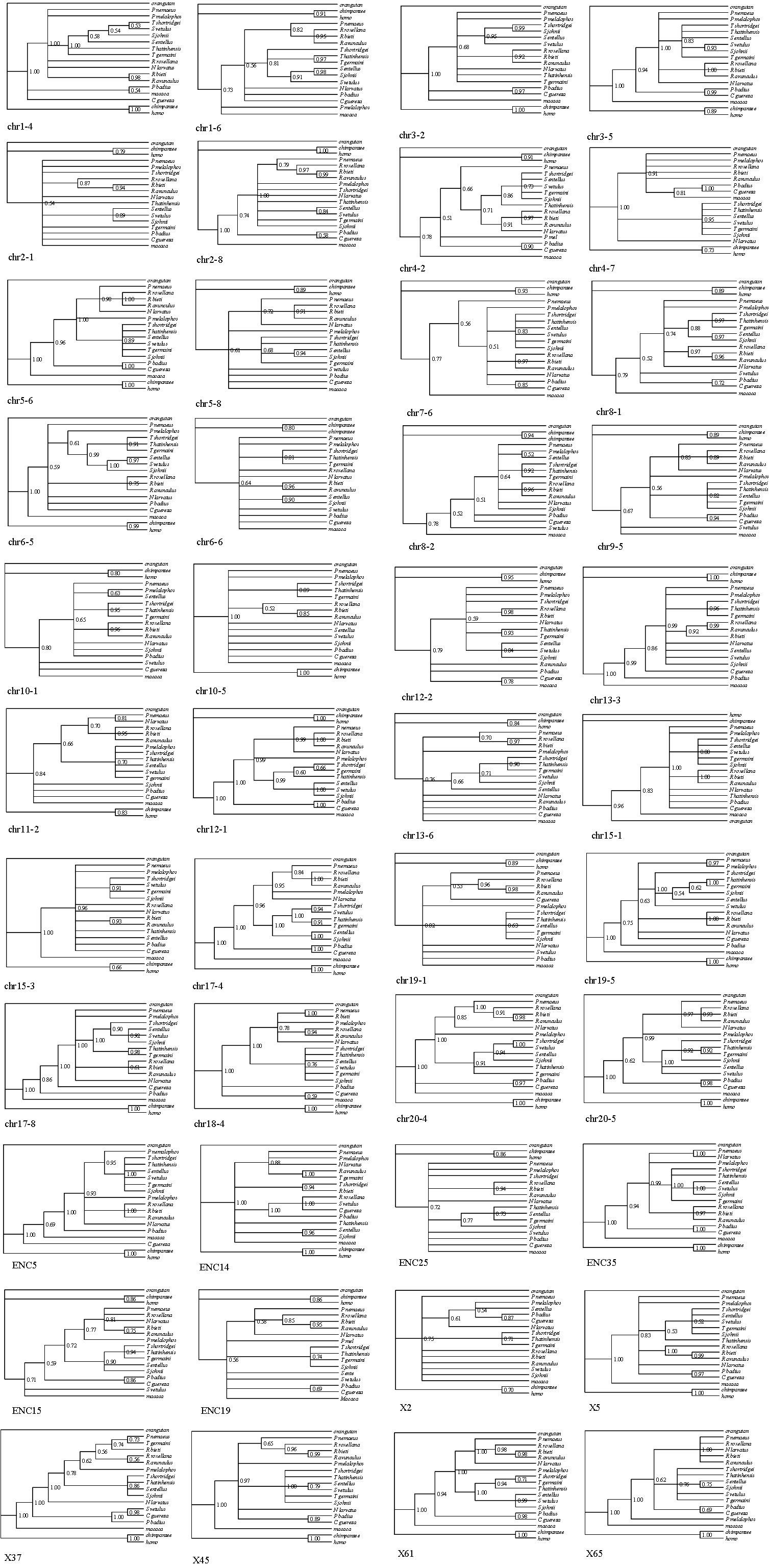

Supplement: Figure S1 — Bayesian trees of the individual nuclear non-coding genes. PPs are presented above nodes. (TIF) [file pone.0036274.s001.tif]

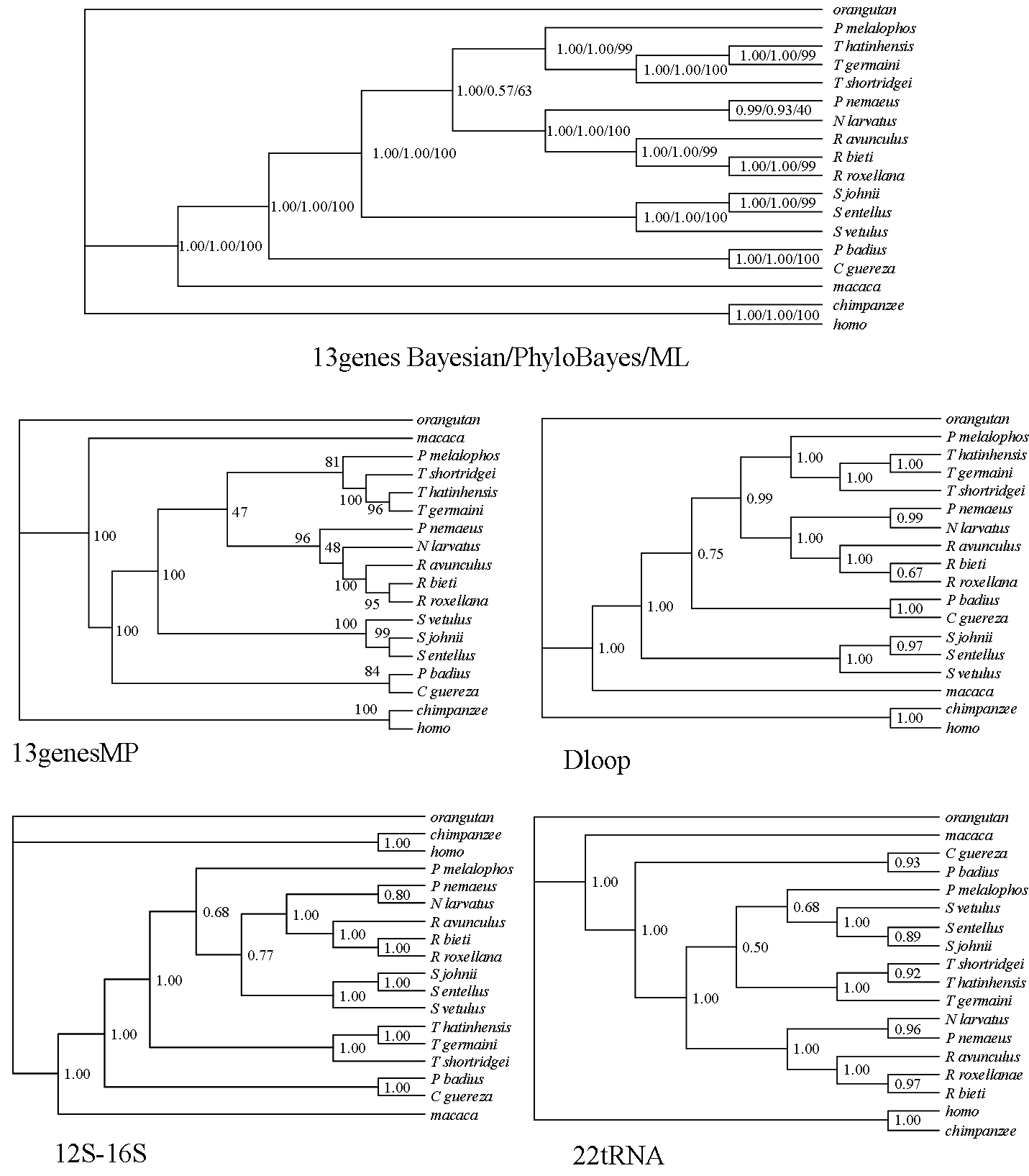

Supplement: Figure S2 — Bayesian trees of the mt datasets (1)–(4). PPs are shown above nodes. Since the resulting tree topologies for the 13 combined protein-coding gene data set differ among the MP/Bayesian/PhyloBayes/ML analyses, trees from all four reconstructions are shown. BS values are shown above nodes. (TIF) [file pone.0036274.s002.tif]

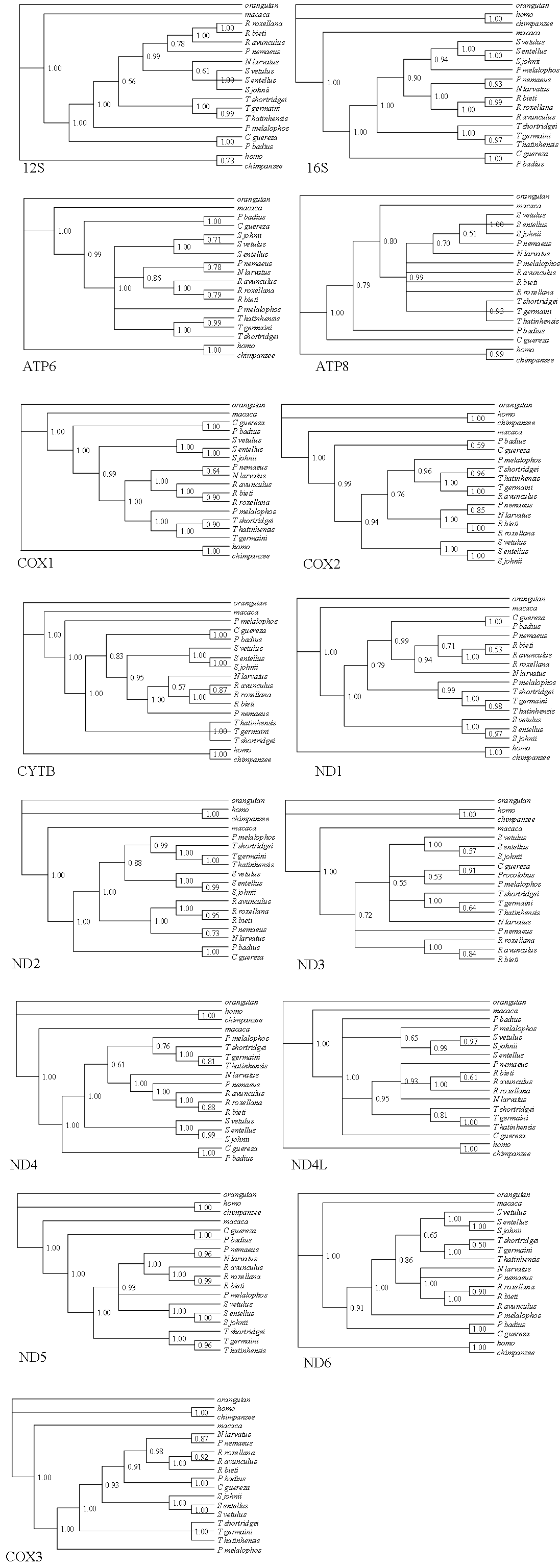

Supplement: Figure S3 — Bayesian trees of the individual mt genes. PPs are shown above nodes. (TIF) [file pone.0036274.s003.tif]
