# Supplementary material for: Phylogenetic Relationships among the Colobine Monkeys Revisited: New Insights from Analyses of Complete mt Genomes and 44 Nuclear Non-Coding Markers
Source: PLoS One. 2012 Apr 27;7(4):e36274. doi: 10.1371/journal.pone.0036274 (PMC3338693; doi:10.1371/journal.pone.0036274)
Supplement: Table S3 — Comparison of phylogenetic performance of mt genes between our study and previous studies. (DOC) [file pone.0036274.s006.doc]

**Table 3**

Comparisons of phylogenetic performances of mt genes among studies.

G, M, P stand for good, medium, poor performance

- stands for no performance evaluation in the study

CR stands for control region, tRNAs stands for combined tRNAs
